# Surgical Management of Traumatic Cervicothoracic Junction Spondyloptosis Without Neurological Injury: A Case Report and Review of the Literature

**DOI:** 10.7759/cureus.30813

**Published:** 2022-10-28

**Authors:** Hani N Alharbi, Ghadeer A Alsager, Mohammed Abdulaziz, Rafiq Bhat, Saad Surur

**Affiliations:** 1 Orthopaedic Surgery, King Saud Medical City, Riyadh, SAU; 2 Orthopaedics, King Saud Medical City, Riyadh, SAU; 3 Orthopaedics and Trauma, King Saud Medical City, Riyadh, SAU

**Keywords:** cervicothoracic, intact, trauma, spondylolisthesis, case report

## Abstract

Acute traumatic cervical spondyloptosis in neurologically intact patients is uncommon and involvement of the cervicothoracic junction is rare. Herein, we report a case of traumatic C7-T1 spondyloptosis in a 56-year-old neurologically intact male patient, with radiographic findings of C7-T1 grade V traumatic listhesis associated with C7 floating segment, cord compression with myelomalacia, extensive ligamentum injury, and intervertebral disc traumatic change and protrusion. He underwent global spine fixation starting with a posterior approach. Follow-up at six months showed good outcomes. The patient was neurologically intact and pain-free; radiographs showed well-maintained fusion and alignment. Controversy surrounds the management of cervical fracture dislocation from all aspects, from "when" to "what." This is the first case reporting a 540° posterior-anterior-posterior approach with successful outcomes. The rarity of cervical spondyloptosis without neurologic injury complicates the management approaches. As few cases are reported in cervicothoracic spondyloptosis literature, it is important to report the present case.

## Introduction

Sub-axial cervical spine facet dislocation can be defined as the anterior translation of the third to seventh vertebrae relative to the inferior vertebra due to unilateral or bilateral joint dislocation [1]. This pattern of injury is mainly attributed to flexion-distraction injury due to motor vehicle collisions [1-2]. Complete spinal cord injury and quadriplegia have been reported with a 50%-84% incidence among bilateral facet dislocated patients [2].

Traumatic cervical fracture dislocation has been described in the literature with varying degrees of listhesis and neurologic injury [2,3]. Among those, cervical spondyloptosis is the most severe type of spondylolisthesis, which when associated with significant neurological deficit leads to devastating consequences, including quadriplegia, respiratory disorders, vertebral artery injury, and death [4]. It is a highly unstable three-column injury that requires immediate reduction and stabilization [5]. Rai et al. reported a total of 47 cases of traumatic cervical spondyloptosis with a 19% incidence of complete cord injury and an American Spinal Injury Association (ASIA) grade A classification, up until 2021 [5].

Moreover, grade V cervical spondylolisthesis in the lower cervical spine is a rare entity and the encountered cases are published as case reports [3]. Among those, only 12 cases described traumatic cervicothoracic spondyloptosis without any neurological deficit/ASIA grade E within the English literature [6-14]. To date, there is no consensus on the management of these complicated cases [5,12].

Herein, we report a case of C7-T1 spondyloptosis in a neurologically intact patient. We aimed to review the management options of spondyloptosis reported in the literature and propose a different management approach based on our specific patient factors.

This manuscript has been prepared in accordance with the EQUATOR Network's research reporting guidelines "CARE" [15].

## Case presentation

A 56-year-old male, with no known pre-existing medical conditions, was transported to our emergency department from a peripheral hospital after being involved in a motor vehicle collision. He was driving the vehicle and was restrained at a speed of 120 km/h. The vehicle did not roll over, and there was no ejection, or fatalities occurred at the scene. The patient presented with neck, back, and chest pain. There was no history of loss of consciousness, numbness, or weakness.

Upon arrival, the patient was awake and alert with a Glasgow Coma Scale (GCS) of 15/15, and was vitally stable. The ABCDE assessment was conducted by an emergency physician. He had multiple facial and skull abrasions and sutured wounds from the referee hospital. He was wearing a rigid cervical collar. Clinical examination revealed a full symmetric motor strength (M5/5), full symmetric sensory examination (S2/2) in all four extremities, a normal rectal tone, and normal reflexes.

Initial investigations

**Figure 1:**
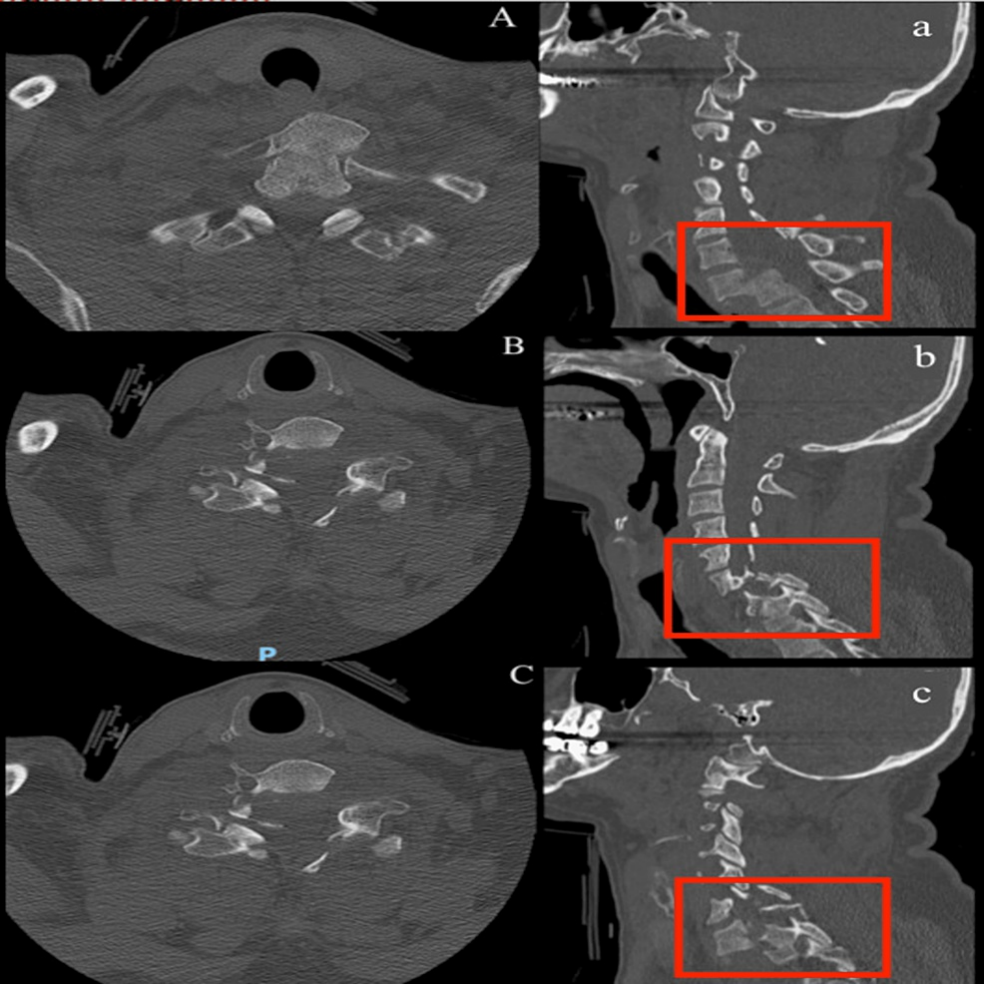
Preoperative computed tomography scan. Sagittal (a,b,c) and axial (A, B, C) views of computed tomography scan showing midcut (A,a), right (B,b), and left (C,c) facet fracture dislocation.

Computed tomography (CT) scans were obtained for the head, whole spine, chest, abdomen, and pelvis. CT of the spine showed forward displacement of C7 over T1, fracture of pedicles, left lamina, and right transverse processes of C7, C6-C7 fracture dislocation, fracture of C6 laminae, pedicles and spinous processes, and locked right facet joint (Figure 1).

Other non-spinal injuries the patient sustained included subgaleal hematoma with surgical emphysema, displaced fracture of the sternum with retrosternal hematoma, bilateral first rib fractures along with the left second rib with lung contusions, and bilateral pleural effusion with dependent atelectatic changes. A CT angiogram of the neck was subsequently conducted. This was unremarkable with patent arteries and no gross stenotic segments, filling defects, or aneurysmal dilatation.

Initial management

The patient was admitted to the intensive care unit for close observation. Although the National Acute Spinal Cord Injury Study (NASCIS III) recommends the use of methylprednisolone [16], our patient did not receive any according to our hospital protocol. The chest injuries were treated conservatively by thoracic surgery. Following this, a neurosurgeon also conservatively treated the brain injuries. For the spinal injuries, the patient was fitted with a Philadelphia collar, with bed rest and spine precautions. As surgical management was planned for the patient, magnetic resonance imaging (MRI) was performed as part of the preoperative planning.

**Figure 2:**
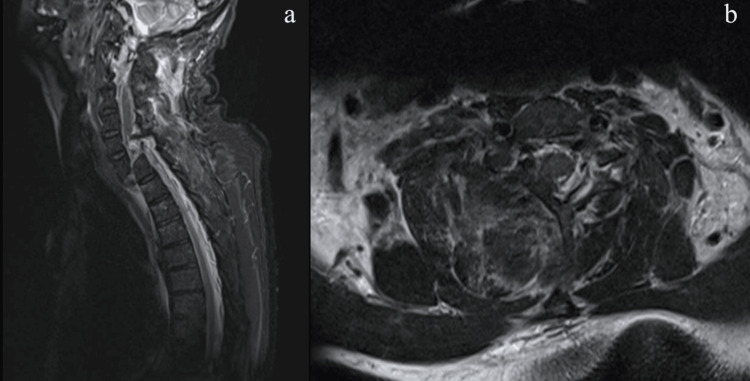
Preoperative sagittal and axial T2-weighted MRI. Preoperative sagittal and axial T2-weighted magnetic resonance images with sagittal (a) and axial (b) cuts showing significant cord compression and complete lithesis of C7-T1 with C7 disc herniation.

MRI showed C7-T1 anterior listhesis (grade V) with left lateral fracture translation in the coronal plane, an associated intervertebral disc traumatic change and protrusion, anterior and posterior longitudinal ligaments, ligamentum flavum, facet joints capsule, and interspinous ligaments disruption with associated high-grade injury of the supraspinous ligament at the nuchal ligament at C5 and T2 levels. Edematous cord changes extended from the C5-T1 levels. T12, T3, and T4 upper-end plate bone marrow contusions were also noted (Figure 2). Consequently, 540° posterior-anterior-posterior spine fixation for cervical injuries and conservative treatment for thoracic injuries was planned.

Surgical procedure

The surgery was carried out under the guidance of fluoroscopy, neuromonitoring, and cranial stabilization using a skull tong traction. Under general anesthesia, the patient was initially placed in the prone position with a standard posterior midline approach. Subcutaneous dissection and exposure of posterior elements from C4-T4 were performed. Next, preliminary lateral mass screw insertion was performed through C3-C4, and pedicle screw insertion through the third and fourth thoracic vertebrae, followed by laminectomy of C7 and decompression, along with a total posterior release. Cerebrospinal fluid (CSF) leakage was visualized from a dural defect. The fragments going to the dura were removed from the right side and the dura was sealed, followed by bilateral C7-T1 facetectomy, which allowed realignment of the spine. No attempt at fracture reduction was made. Next, the patient was turned into the supine position, and a left-sided anterior Smith-Robinson approach was utilized for C7 corpectomy, C7-T1 discectomy, and C7-T1 junction release, which was followed by spontaneous reduction. The C6-T1 plating was fixated with screws and the mesh cage and bone graft were applied between C6-T1. The T1 body was intact and held the fixation well, which omitted the need for manubriotomy. Lastly, the patient was turned to the prone position again and the cervicothoracic components were connected with a 3.5 mm cervical rod and a 5.5 mm thoracic rod with a crosslink in-between. The wound was closed in layers. The patient tolerated the surgery well, was extubated, and placed in a cervical collar.

Postoperative course

**Figure 3:**
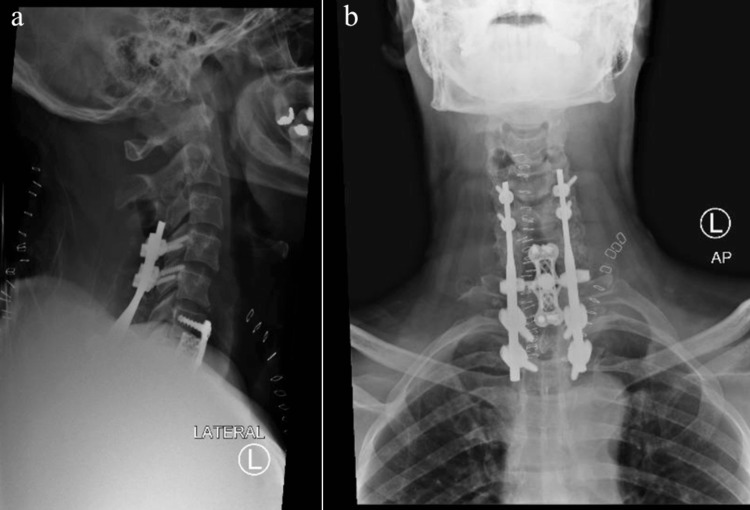
Postoperative anterior-posterior and lateral X-ray images. Immediate postoperative plain radiographs showed good realignment and correct screw placements

The patient performed well during recovery and was neurologically intact. Postoperative lateral and anterior-posterior plain radiographs showed good realignment and correct screw placements (Figure 3). The postoperative period was uneventful and there were no surgery-related complications. The patient mobilized with physical therapy and was discharged on the tenth postoperative day. He remained in a cervicothoracic brace for six months.

Follow-up

**Figure 4:**
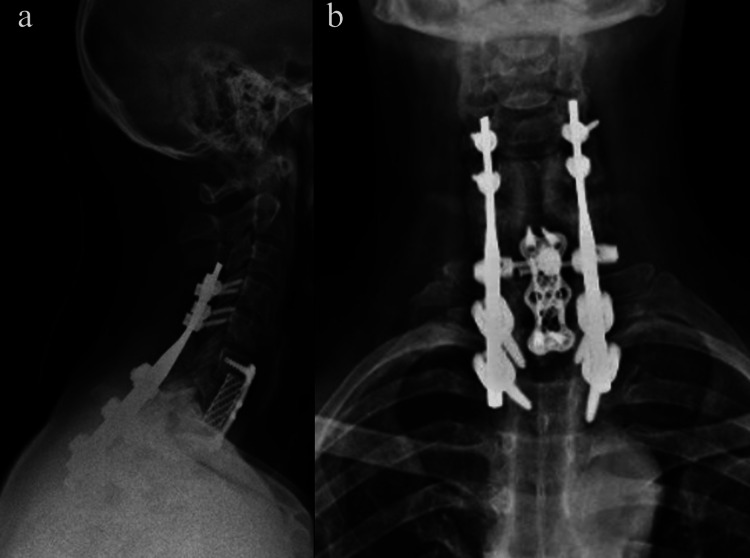
Follow-up anterior-posterior and lateral X-ray images at three months. Follow-up cervical X-ray images showing maintained reduction and alignment, and good fusion.

**Figure 5:**
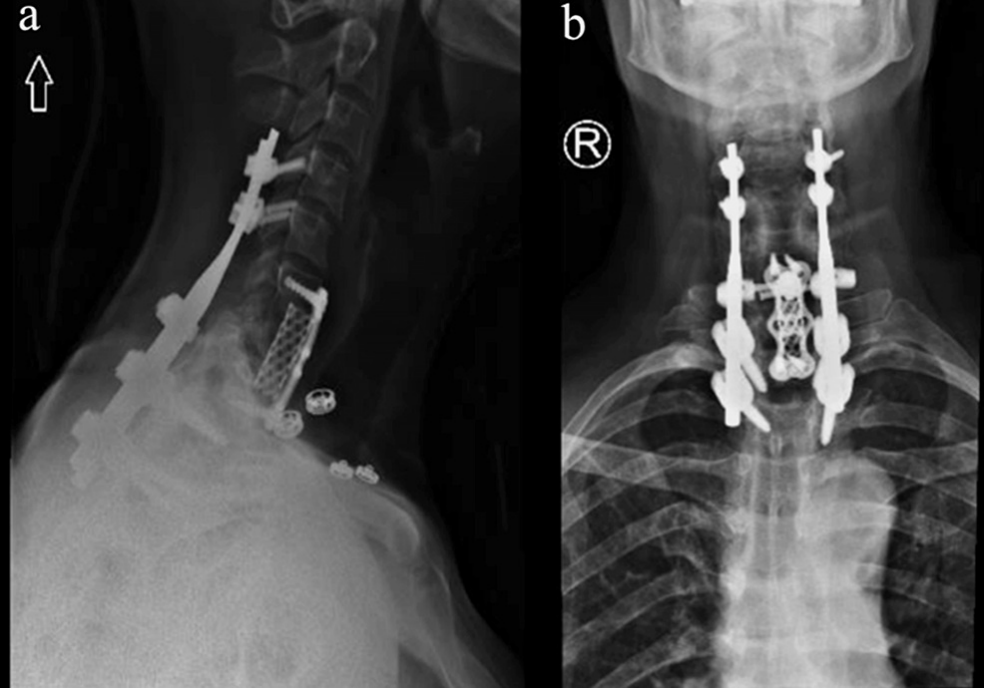
Follow-up anterior-posterior and lateral X-ray images at six months. Follow-up cervical X-ray images showing maintained reduction and alignment, and good fusion.

At the six-month follow-up, the patient remained asymptomatic and did not present with any functional limitations. Functional outcomes were assessed using Vernon and Mior's 5-point-scale ("Neck disability index") [17] Our patient scored 4, which is interpreted as no disability. Follow-up cervical X-ray images showed maintained reduction and alignment, and good fusion (Figure 4-5).

## Discussion

Traumatic cervical spondyloptosis is a complete cervical spine vertebral body displacement relative to the inferior vertebra body, which requires urgent management to avoid fatal consequences [5]. There is an ongoing debate regarding the management of cervical spondyloptosis, as many factors should be considered before a management plan can be unanimously decided upon. These disagreements range from initial investigations to definitive management plans. The patient's neurological status, the severity of radiographic findings, and the reduction status of the dislocation are the most important factors to consider [5,12,18]. Moreover, the rarity of C7-T1 spondyloptosis without neurologic injury further complicates the management approach since there are few reported cases in the literature of cervicothoracic spondyloptosis [6-14]. A summary and comparisons of the literature on acute traumatic cervicothoracic (C7-T1) spondyloptosis among neurologically intact/ASIA E adult patients are shown in Table 1.

**Table 1:** Summary and comparison of the literature on acute traumatic cervicothoracic (C7-T1) fracture-dislocation with spondyloptosis among neurologically intact/ASIA grade E adult patients​​​​​​​ MOI: mechanism of injury; F/U: follow up; MVC: motor vehicle collisions; F: female; M: male; Lt: left; Rt: right; NA: not available; ACDF: anterior cervical discectomy and allograft fusion; PSF: posterior spine fixation; A: anterior; P: posterior; CSF: cerebrospinal fluid; GA: general anesthesia *Outcomes = In term of (1) Examination: Neurologically intact, (2) Imaging: Reduction maintained, well aligned, corrected and fuse

Reference	Age	Sex	Fractured element	MRI findings: 1-Disc prolapse? 2-Cord injury?	MOI	Initial management (Traction?)	Reduction successful?	Definitive management	F/U (months)	Outcomes*
Current Case	56	M	C6 Laminae + Pedicles, C7 Lt Lamina + Pedicles	1-Yes; 2-Yes	MVC	No	___	540 ° (P+A+P)	6	Good
Tumialán et al. [6]	48	M	C6 Facets + Pedicles, C7 Laminae + Pedicles	1-Yes; 2-No, CSF signal	MVC	Yes (27 kg)	Yes	360 ° (A+P)	12	Good
Acikbas and Gurkanlar [7]	42	M	C6-T1 Lamina +Lateral mass	1-No; 2-No	MVC	Yes	Yes	2-Stages (ACDF →PSF)	NA	No neurologic deficit
Munakomi et al. [8]	56	F	C7 Laminae + locked facet	No MRI	Fall	Yes	No	Manual reduction under GA → 540 ° (A+P+A)	NA	Immediate post-operative: Good
Kumar et al. [9]	40	M	C7 Pedicles, C6/C7 unilateral facet	1-Yes; 2-No	MVC	1-No	___	540 ° (A+P+A)	12	Good
Nguyen et al. [10]	63	F	C6-C7 Lt Locked facet, C7-T1 Rt Locked facet, C7 Lt Pedicles + Rt Lamina	1-Yes; 2-Yes, minimal edema	MVC	Yes (7 kg)	Partial	360 ° (A+P)	3	Good
Nguyen et al. [10]	60	M	C7 Pedicles + Laminae	1-Yes; 2-No	Bicycle collision	Yes (4.5 kg) Tongs →Halo	No	2-Stages (ACDF → PSF)	6	Good
Lee et al. [11]	72	M	Posterior element and bilateral locked facet	1-Yes; 2-Yes, cord hyperintensity	Fall	1-No	___	Rigid neck collar	3	No neurologic deficit
Dahdaleh et al. [12]	51	M	Bilateral locked facet	1-No; 2-No	MVC	Yes (20 kg)	Partial	PSF	12	Good
Ahn et al. [13]	32	M	C7 pars and bilateral locked facet	1-Yes; 2-No	Fall	Yes (27 kg)	No	2-stages (ACDF → PSF)	___	Immediate post-operative: Good
Ahn et al. [13]	42	F	C7 pars	1-Yes; 2-No	Fall	Yes (18 kg)	Yes	360 ° (A+P)	___	Immediate post-operative: Good
Payne et al. [14]	63	M		1-No 2-No	Fall	No	___	PSF	5	Good

Management approaches range from single versus double versus triple surgical options, either within the same session or more than one session, with preoperative reduction or without [5]. Most cases reviewed in the literature preferred a combined approach [6-10, 13].

We also elected a combined approach, without preoperative traction, due to the unstable nature of the injury and the presence of disc prolapse, using a posterior-anterior-posterior approach. A complete release of the cord was performed posteriorly without any reduction, followed by anterior release with C7 corpectomy, discectomy, and release of C7-T1 junction, which achieved a spontaneous reduction. Fusion of C6-T1 was performed followed by posterior spine fixation from C4-T3.

Among the cervicothoracic spondyloptosis case reports reviewed, Munakomi et al. [8] and Kumar et al. [9] utilized a similar 540° fixation, but with an anterior-posterior-anterior approach. The choice of an anterior approach for discectomy was reasoned by the presence of disc prolapse, which could cause further damage if the patient is placed in the prone position initially [8-9]. Bartels and Donk [18] described the delayed diagnosis of a cervicothoracic fracture dislocation in three patients. In the first two patients, they planned for a traditional 540° spine fixation approach starting anteriorly, after anterior discectomy and a failed reduction trial. Although they proceeded posteriorly, they could not achieve good aliment or reduction; therefore, eventually, after anterior reduction in the third position, they had to add a posterior fourth stage for final fixation. To reduce the operative time with its potential risks, they proposed the following sequence: posterior facetectomies without a reduction trail, followed by an anterior discectomy with reduction of the dislocation and anterior fixation, and finishing with posterior fixation. They reported a third case utilizing this approach with successful outcomes [18]. Ozdogan et al. justified starting posteriorly to avoid a possible graft displacement if it was placed first [19].

In our patient, due to C7 severe disc prolapse and the presence of an unstable C6-C7 posterior element fracture with the fragments indenting the cord, an anterior approach was avoided to prevent endangering the cord, and a posterior decompression without reduction was attempted first.

Several authors proposed an algorithm for the treatment of cervical spondyloptosis to aid in decision-making when facing such an uncommon case [5,12,20]. After obtaining CT and MRI images, Dahdaleh et al. based their approach on the findings relating to anterior cord compression; if present, an anterior approach was elected, whether combined or not, it depends on the intraoperative reduction status and the stability of reduction [12]. Padwal et al. suggested a similar treatment algorithm, but this depended on the preoperative traction along with the anterior cord compression [20]. Rai et al. also suggested a similar algorithm; however, they considered the patient’s consciousness, neurologic grade, and reduction status [5].

Although these studies offer a valid and reasonable algorithm, due to the many factors to be considered in the final decision, none have been validated so far. Moreover, an important factor they failed to incorporate is the extent and severity of the fracture, which might prevent initiating an anterior approach to avoid posterior compression [5,12,20]. Until an adequate number of cases have been reported and a unified treatment algorithm has been validated, we believe that each case should be tailored individually, considering what has been reported thus far.

From our experience, we recommend careful selection of the appropriate surgical intervention when dealing with such cases, considering all potential factors. When there is extensive damage to the posterior elements, a posterior-anterior-posterior approach is recommended to avoid posterior compression from a fractured segment. However, further cases should be reported to support our findings.

This study is limited by the short follow-up and reporting outcomes from a single patient.

## Conclusions

Cervical spondyloptosis in a neurologically intact patient is a rarely encountered case and poses a management dilemma. A global fixation is recommended due to the instability of the fracture. We used 540° fixation with a posterior-anterior-posterior approach with successful outcomes and recovery compared to the anterior-posterior-anterior approach classically utilized. We recommend taking into account the extent and stability of the posterior element fracture when considering the management approaches in similar cases.
